# Universal health coverage and equal access in Sweden: a century-long perspective on macro-level policy

**DOI:** 10.1186/s12939-024-02193-5

**Published:** 2024-05-28

**Authors:** Mio Fredriksson

**Affiliations:** Department of Public Health and Caring Sciences, Health Services Research, Uppsala University, Box 564, Uppsala, 751 22 Sweden

**Keywords:** Universal health coverage, Equal access, Sweden, Swedish model, Horizontal equity, Vertical equity

## Abstract

**Background:**

When today’s efforts to achieve universal health coverage are mainly directed towards low-income settings, it is perhaps easy to forget that countries considered to have universal, comprehensive and high-performing health systems have also undergone this journey. In this article, the aim is to provide a century-long perspective to illustrate Sweden’s long and ongoing journey towards universal health coverage and equal access to healthcare.

**Methods:**

The focus is on macro-level policy. A document analysis is divided into three broad eras (1919–1955; 1955–1989; 1989–) and synthesises seven points in time when policies relevant to overarching goals and regulation of universal health coverage and equal access were proposed and/or implemented. The development is analysed and concluded in relation to two egalitarian goals in the context of health: equality of access and equal treatment for equal need.

**Results:**

Over the past century, macro-level policy evolved from the concept of creating access for the neediest and those reliant on wages for their survival to a mandatory insurance with equal right to healthcare for all. However, universal health coverage was not achieved until 1955, and individuals had to rely on their personal financial resources to cover the cost at the time of care utilization until the 1970s. It was not until 1983 that legislation explicitly stated that access to healthcare should be equal for the entire population (horizontal equity), while a vertical equity-principle was not added until 1997. Subsequently, ideas of free choice and privatization have gained significance. For instance, they aim to increase service access, addressing the Swedish health system’s Achilles’ heel in this regard. However, the principle of equal access for all is now being challenged by the emergence of private health insurance, which offers quicker access to services.

**Conclusions: brief summary and potential implications:**

It can be concluded that there is no perpetual Swedish healthcare model and various dimensions of access have been the focus of policy discussion. The discussion on access barriers has shifted from financial to personal and organizational ones. Today, Sweden still ranks high in terms of affordability and equity in international comparisons: although not as well as a decade ago. Whether this marks the beginning of a new trend intertwined with a decline in Sweden’s welfare ‘exceptionalism’, or is a temporary decline remains to be assessed in the future.

## Introduction

At present, 30% of the of the world’s population do not have access to essential health services [1] and one of the greatest inequalities globally lies in the unequal access to safe, effective health care without financial hardship, i.e. *universal health coverage* [2]. Universal health coverage is a strategic priority for The World Health Organization (WHO) and “means that all people have access to the full range of quality health services they need, when and where they need them, without financial hardship”. This applies to essential services such as health promotion, prevention, treatment, rehabilitation as well as palliative care [3]. It is built on the foundations of human rights and equity, with a commitment to providing health for all and reducing inequalities [4]. According to the WHO, paying for healthcare out of pocket in case of unexpected illness may push people into poverty or destroying their future, or that of their children; and protecting people from those financial consequences is thus of great importance [3]. This is associated with one of the three objectives of universal health coverage: improving financial protection, where avoidance of direct payment at the point of care utilization is essential [5]. The other objectives include improving equity in the utilization of needed services and improving service quality [6].

Every country has a different path to achieving universal health coverage, influenced by factors such as the health needs of the population, the level of resources available, and the political and legal environment. Drawing from literature on the evolution of welfare states, diversity in goals, timing, public-private mix etcetera has been attributed to various factors, including different legacies related to state and nation building, trust in governmental capacity to address issues, and societal divisions [7]. Consequently, some countries face significantly greater challenges than others in their efforts to achieve universal health coverage, such as those stemming from issues like corruption. Political leadership has however been established as especially important [2]. To make health a reality for all it is essential with policy-makers committed to investing in universal health coverage and equity-oriented policies [3]. However, even under favourable conditions, achieving this goal may take some time. When today’s efforts to achieve universal health coverage are mainly directed towards low-income settings, we may forget that countries known for having universal, comprehensive and high-performing health systems—who might be considered role models—have also undergone this journey, which remains ongoing. This includes Sweden, which is an oft-mentioned model country in terms of universal health coverage, having a tax-funded health system with low out-of-pocket payments and overall goals of equal access for all [8]. However, this has not always been the case, and Sweden’s health system (being of National Health Service type [9]) is very different today compared to the time when the pursuit of universal health coverage began over a century ago. Moreover, the challenges today also differ from those at the beginning of the twentieth-century.

In this article, the aim is to provide a century-long perspective to illustrate Sweden’s long and ongoing journey towards universal health coverage and equal access to healthcare. The focus is on how macro-level policy with significance for universal health coverage and equal access in has developed over the past 100 years. Providing such a long-term perspective is important to understand that health systems are influenced by events in the surrounding society, and that some policies may need repeated attempts. Furthermore, it is essential to recognize that no solutions are permanent but that policy is constantly evolving. It also highlights the importance of political will and resolve, exemplified in this case by the prolonged government tenure of the Social Democrats, characterized by a dedication to comprehensive social policies and equality. This culminated in the Swedish welfare state model [10], with recent developments by center-right parties rather emphasizing the timeliness and quality of care. The theoretical point of departure in the article is Abatemarco, Beraldo et al.s [11, p. 14] proposition that reducing inequality in opportunity to access healthcare “first requires granting universal access and then equalizing conditions for access”. This means that a first step toward improving equity in the utilization of needed services is to close the gap between individuals having access to health treatments and those who have not. Hence, the first of the tree eras portrayed in this article describes the struggle for a compulsory health insurance granting access to healthcare for all, which was not achieved until 1955. The two latter eras aim to capture different types of macro-level policy with significance for equal access. As we shall see, over the past century, during which life expectancy has risen from 57 years to 83 years, various aspects of access have been at the forefront of policy development. This includes considerations on how to ensure equal access and mitigate potential challenges, with access barriers shifting from financial barriers to personal and organizational ones. Notably, individuals had to rely on their personal financial resources for direct payment at the point of care utilization until the 1970s, and it was not until 1983 that legislation *explicitly* stated that access to healthcare should be equal for the entire population (horizontal equity). That those with the greatest need for healthcare should be given priority did not enter into legislation until 1997, adding a vertical equity principle as well. Overall, this shows a gradual increase in measures to achieve equal access, which might be challenged by reforms during the current era of choice and privatization.

Even though this article spans a century, prior to that, there was a mixed economy of welfare, where the state was one among several actors in a system that included voluntary and informal welfare providers [12], with third-sector organizations arranging, for example, voluntary sickness insurance programs [13]. Some social insurance decisions were already made at that time: in 1913, a general pension insurance (covering old age and invalidity), and a few years later, in 1916, an accident insurance that covered almost the entire labor market. Research on the development of the Swedish welfare state has, for example, examined mutual health insurance in early twentieth-century Sweden and workplace accident insurance among workers [14]. However, this research has almost exclusively focused on the sickness absence aspect of health insurance [14, 15]. Moreover, there exists a vast literature on the idea of the welfare state (an expression originating from Britain [16]), and its expansion in Europe and the United States. This literature highlights the welfare state as the outcome of a combined political and economic development intertwined with democracy and industrialism [17]. Not least, Esping Andersen [18] emphasizes the importance of historical characteristics and that states prioritize differently, resulting in welfare states of various types—Sweden being of ‘social democratic’ type. Rothstein [19] argues that, in a comparative perspective, the Swedish welfare state “has generally been seen as more encompassing, more universalistic, and more redistributive than other welfare state systems”. However, the focus of this article is much narrower, with its emphasis on the development of the ‘health care state’ [20], which followed a different trajectory compared to, for example, the National Health Service (NHS) in England, founded in 1948 as a comprehensive health service, free at the point-of-delivery, available to everyone, and funded through general taxation [21]. During the period under examination in this article, most advanced industrial countries came to guarantee healthcare services for their populations through some public scheme, albeit ‘by different routes, in different ways, using different sorts of institutional apparatus’ [20]. Here, we take a closer look at Sweden.

The paper is structured as follows. First, a theoretical elaboration of two egalitarian goals in the context of health is provided: equality of access and equal treatment for equal need [22]. These egalitarian goals are helpful to understand the long-term policy development regarding universal health coverage and equal access in Sweden. Thereafter, empirical sources and delimitations are presented in the Methods section and a brief description of how the Swedish healthcare system is structured is provided. The analysis is divided into three broad eras (1919–1955; 1955–1989; 1989-) and focuses on seven points in time during the past century when macro-level policy with relevance for overall goals and regulation of access have been proposed and/or implemented in Sweden. The policy development between these seven points in time is summarised. Overall, the three broad eras correspond to the four latter stages in the evolution of the welfare state in Europe: the introductory phase (1880–1914); the expansion phase (1914–1945); the consolidation phase (1945–1953); the ‘golden age’ (1953–1971) and the retrenchment phase/’silver age’ (1971–2000) [7, 23]. The article concludes with an analysis and discussion of the main trends from the past 100 years in relation to equality of access and equal treatment for equal need. By discussing the development based on these egalitarian goals, comparisons with the long-term evolution of other healthcare systems can be made more easily.

### Theory: egalitarian goals in the context of health

Based on relevant literature in the field, the theory section elaborates on two types of egalitarian goals in the context of health: (1) equality of access and (2) equal treatment for equal need [22]. These goals will structure the discussion of the results, which are presented chronologically to maintain the narrative.

***Equality of access***. According to Le Grand, equality of access can be defined as the requirement that individuals should face the same personal cost of receiving medical treatment. Inequality of access exists, for example, if some people have to travel further, face higher personal cost, are required to wait longer or are charged more than others for treatment [22]. Inequality also exists if the medical treatment different groups can access is of different quality [24] and some are left to poor-quality services [25]. Abatemarco, Beraldo et al. [11] argue that to address access disparities we need to consider both disparities between individuals who have access to healthcare and those who have not, and between individuals having access. About why access is so important, Culyer [26] argues it is important for needs to be assessed, and then met equitably.

In effect, access depends on a combination of factors. Rodriguez Santana, Mason et al. [24] argue that, at the macro level—access refers to the population having the opportunity to use healthcare services (‘having access’): which is as supply-side definition of access. At the micro level, it is about individuals ‘getting access’. Utilization of services is created in the intersection between need and/or demand and supply. Some scholars have further detailed different dimensions of access, see for example Levesque, Harris et al. [25] proposing the dimensions of approachability, acceptability, availability and accommodation, affordability and appropriateness. Making a partially different division, Gulliford, Figueroa-Munoz et al. [27], argue that the first important dimension is *service availability*, i.e. that there is an adequate supply of services available, measured e.g. as doctors per capita but also as timeliness of care. This may vary due to geographical conditions, creating e.g. longer travel distances and higher cost in some areas. Furthermore, Gulliford, Figueroa-Munoz et al. [27] discuss *utilization of services and barriers to access*: there may be formal service availability, but people may encounter difficulties utilizing the services. They mention three barriers. The first is *personal barriers* (approachability and acceptability): i.e. individuals’ perceptions of their needs, their attitudes and beliefs, and previous experiences influence the probability of using services. Social and cultural influences are also of importance. The second is *financial barriers* (affordability). Even if a service is free at the point of use, there may still be costs for additional services. Costs may also be due to time lost from work or travel expenses, which affect different socioeconomic groups differently. For some groups, even small costs make people abstain from seeking care. Thus, equal costs do not necessarily give equal access. In line with this, Abatemarco, Beraldo et al. [11] stress that a key disparity between those who have access to a service and those who have not, may be costs. Costs may also be a barrier to access high quality services (appropriateness). The third barrier is *organizational barriers* (availability and accommodation), which may be long waiting lists, referral practices, inefficient use of capacity or failure to design services around the needs of patients.

***Equal treatment for equal need***. Le Grand argues that this goal is intuitively appealing, but points to the difficulty of defining what treatment is (and if equality refers to quantity, expenditure, value for the individual etc.) as well as what need is. Many others have discussed how to define need. Culyer [26, p. 278], for example, maintains that “a need for health care can exist only when there is a capacity to benefit” from a treatment or intervention. Thus, he concludes that need is not synonymous to ill health, although this is sometimes assumed.

Further, Rodriguez Santana, Mason et al. [24], point out that it is a difference between need for health and need for health care. Need for health (where healthcare plays a part) is usually explained by what it is able to accomplish; it is usually pictured that “good health is necessary for a person to flourish” [24, p. 1]. “One cannot ‘flourish’ at all if one is dead or diseased” [26, p. 276] and to a limited extent if being ill or injured. Therefore, unmet need is important to handle. Rodriguez Santana, Mason et al. [24] specify five types of unmet need: *unperceived* (by the individual); *chosen* (informed); *unchosen* (because of external factors such as lack of staff and unaffordable travel costs); *clinician validated* (does not receive care a clinician would consider appropriate) and *subjective unmet expectations* (based on the individual’s view of the appropriateness of treatment).

In relation to need, the principle of horizontal equity means that “like needs should receive like attention and resources” and the principle of vertical equity “that greater needs should receive greater attention and resources” [26, p. 276]. In most systems, there is however also a discussion regarding whether ‘merit’ is relevant and if people should be prioritized based on contribution to society (e.g. to those who are breadwinners or have children) or down prioritized or asked for additional payment based on negligence or negative lifestyle choices (e.g. bad eating habits, drinking, smoking, sedentary lifestyle) [26]. In the literature, there is, however, a discussion on how autonomous a person’s choice is and to what extent that choice is constrained by factors beyond a person’s control (e.g. family poverty), also on how autonomous preferences are [22].

## Methods

The empirical focus of this paper is on the macro-level development of universal health coverage and equal access in Sweden. The macro-level development includes among other things legislation and political ideology, in contrast to the organizational level (e.g. economic resources, staff, rule systems) and the tangible/practical level (e.g. what is actually done and how resources are spent) [28].

Thus, the focus is on how the idea of universal health coverage and equal access has been expressed and regulated at the national level. This means that a large number of reforms, some of which may have had an impact on access or distribution of access, are left out. For example, there have been a continuous stream of reforms regarding service organization, e.g. the preferred balance between ambulatory care and hospital care, regulation of private providers, and education policy for doctors and nurses. These may directly or indirectly have affected access at the practical level. In addition, number of doctors, hospitals, waiting-times etcetera are also not systematically covered, but rather exemplified. Nor do I go into detail in the political disputes, and it should be noted that a number of reforms directly affecting health outcomes, such as work environment laws and road safety regulations, are outside of the scope of the article.

The study covers roughly one hundred years and are split into three eras: (1) The era of struggle for a compulsory health insurance (1919–1955); (2) The era of emphasis on equity and public provision (1955–1989); and (3) The era of choice and privatization (1989-). In these eras, seven points in time with relevance for overall goals and regulation of equal access in Sweden are zoomed-in: 1919, 1944, 1955, 1970, 1983, 2010 and 2020, Table 1. To get a sense of the overall development, the main events between these points in time are summarised.

The empirical material consists primarily of primary sources, but secondary sources are used as a complement. The primary sources are principally parliamentary Inquiry reports commissioned by the Swedish government (*Statens offentliga utredningar*, SOU) and government bills (*propositions*) based on these Inquiry reports, Table 1. The process of including empirical sources was incremental and it was not decided in advance which documents were to be analysed. Qualitative document analysis, which is a systematic procedure used for making empirical observations based on written records [29], was used in order to find content of relevance for the macro-level development of universal health coverage and equal access. A qualitative synthesis of the content was made [30].

**Table 1:** Main events covered in the analysis of macro-level policy development

Year	Policy event	Name of Inquiry	Main sources
The era of struggle for a compulsory health insurance (1919–1955)			
1919	Universal healthcare insurance proposed. *Postponed.*	Socialförsäkringskommittén	Betänkande och förslag angående allmän sjukförsäkring, 1919
1944	Universal healthcare insurance proposed. *Postponed.*	Socialvårdskommittén	SOU 1944:15 Prop. 1946:312
1955	Universal healthcare insurance proposed in 1952. *Implemented in 1955.*	Socialförsäkringsutredningen	SOU 1952:39 Prop. 1953:178
The era of emphasis on equity and public provision (1955–1989)			
1970	Cost and funding reform. *Implemented in 1970*.	Sjukförsäkringsutredningen	SOU 1967:63 Prop. 1969:125
1983	New healthcare legislation (1982:763). *Implemented in 1983.*	Hälso- och sjukvårdsutredningen	SOU 1979:78 Prop. 1981/82:97
The era of choice and privatization (1989-)			
2010	Mandatory choice and free establishment of providers in primary care. *Implemented in 2010.*	Utredningen om patientens rätt	SOU 2008:127 SOU 2008:37 Prop. 2008/09:74
2022	Limiting private health insurance’s impact on publicly funded health care. *No measures taken*.	Utredningen om privata sjukvårdsförsäkringar	SOU 2021:80 Ds 2022:15 Betänkande 2022/23:SoU5

### Overview of the Swedish health system

The Swedish healthcare system is of the National Health Service type (Nordic countries, UK, Portugal and Spain), in which regulation, financing and provision are governed by the state. These systems have a commitment to universal coverage, equal access to services and beliefs in the efficiency of public services [9]. The Swedish healthcare system is however more decentralized than e.g. the English NHS and the responsibility for healthcare is divided between three governing levels: the state, the regions (previously county councils) and the municipalities. The 21 regions are self-governing and have the responsibility to finance and provide healthcare to its inhabitants. This responsibility was transferred successively during the past century and finalized in 1983. However, as early as the 1860s the county councils became responsible for somatic hospitals [31]. The hospitals have in principle always treated accidents and acute illnesses [32] and been public. From the late 1930s, the county councils were also charged with non-hospital services such as maternity and paediatric healthcare and they employed district nurses and midwifes [31]. Today the regions levy taxes to fund healthcare, currently about 11–12% of people’s incomes.

A system with district doctors was founded at the end of the 1600s, but there were also city doctors serving the urban population. Up to the beginning of the twentieth-century, the majority of all physicians were general practitioners with a private practice and there were few hospital doctors. However, this changed rapidly during the first half of the twentieth-century when the number of hospital specialists grew swiftly with the fast expansion of the hospitals [33]. The system with district doctors was gradually undermined by hospital specialists providing outpatient care at the hospital (but as their own private practice) until 1958 when it was suggested that single GP practices should be replaced with GP practices with two or more doctors. The coming 30 years this way of delivering primary care was consolidated into a system with rather large primary healthcare centres with a multi-disciplinary team and an area responsibility for the population living in the primary healthcare centre’s area to cover. From 2010, however, private providers have the right to establish a primary care centre with public funding if they meet the requirements set up by the region.

Today, there are seven regional hospitals in Sweden and about 70 county and district hospitals. Only four OECD countries have fewer hospital beds (2.07 per 1000 inhabitants). There is about 1200 primary healthcare centers of which 45% are privately run. There is a maximum payment per year and person for open care in Sweden: ∼114 EUR. In 2021, 11.3% of GPD was spent on healthcare. Regarding healthcare outcomes, Sweden performs well [34] and very few abstain from seeking healthcare due to costs.

### The era of struggle for a compulsory health insurance (1919–1955)

Leaving the introductory phase of the welfare state with an emphasis on voluntary solutions, the expansion phase was entered in 1915, when a non-political government appointed a parliamentary Inquiry (*Socialförsäkringskommittén*) that proposed a universal and compulsory health insurance in 1919. This proposal had broad political support [35]. The Inquiry described the increasing number of workers who were dependent on wages for their survival, an effect of modern industrialization, and how their ability to work could be fully or partially suspended by illness, accidents, or invalidity, leaving them without an opportunity to work and pay for their living [36]. A compulsory insurance was according to the Inquiry necessary because the existing voluntary insurance through sickness funds did not reach the neediest. Since 1881, the state subsidized voluntary sickness funds, but they never got an acceptable number of members [35]. In fact, these sickness funds mainly provided income maintenance insurance while medical care was largely provided on an individual fee-for-service basis [37]. As we shall see, from the first proposal in 1919, it took until 1955 for Sweden to get a universal health insurance, which was much later than their neighbours Norway and Denmark [37]. One of the major debates revolved around the funding, which posed an additional challenge due to the insurance covering not only medical care but also providing cash benefits in case of illness. The primary impetus during this period was to *reduce financial barriers* in the population in accessing healthcare. It is worth noting that physicians were among those exhibiting the strongest opposition to a compulsory health insurance [28, 37, 38]. Primarily, this was rooted in a fear that their position as independent professionals would be weakened and that it would lead to an emerging “medical proletariat” [35, 38].

#### 1919: the first draft of a universal health insurance is presented

The Inquiry proposed that the insurance would include all individuals over 16 years of age with the ability to work, with exceptions for those with an income or assets over a certain sum and those with better health and sickness benefits from an employer [35, 36]. The insurance would cover medical care (*läkarvård*) and medicines for the insured and their children, as well as cash benefits in case of illness (*sjukpenning*). The right to medical treatment and medicines was to be *equal for all insured*, but the right to cash benefits was not. The insurance would only cover healthcare and medicine costs *when there was a need*, and thus not preventive care. However, the Inquiry left the definition of disease/illness to the doctors. Since the aim of the new insurance was to improve general health, healthcare would be provided even if the illness or injury was caused by drinking, indecency, fighting, and so on.

The insurance would be funded by two-thirds of the fees paid by the insured themselves (which could be paid by the employer and deducted from the salary), some risk fees by employers, and about one-third by public funds [35, 36]. It would require a heavy investment of public funds, but the Inquiry argued that an improvement of the general state of health, and the reduction in poverty costs and other expenses for healthcare, justified such sacrifices [36]. However, due to concerns about service availability, the Inquiry warned that implementing the insurance could be difficult because there were currently too few doctors and an uneven distribution of doctors geographically, especially between urban and rural areas.

#### 1920–1940: postponed reform and state-subsidized voluntary sickness funds

The proposed universal and compulsory health insurance had strong political support, primarily expressed by the Social Democrats showing an increasing commitment to socialised medicine [35, 37]. However, due to economic recession, deflation crisis, and high unemployment during the 1920s, it had to be postponed [39, 40]. Arguments for savings on government spending during this period were voiced primarily by the liberal and conservative parties, but no parties protested loudly against postponing the universal insurance [35]. The solution became to develop the existing system with state-subsidized voluntary sickness funds, and in 1931, monopoly sickness funds were introduced, providing cash benefits in case of illness and compensation for medical care [35, 41, 42]. At most, two-thirds of the population was registered with a sickness fund [42], in 1930 about 20% of the adult population [43]. During the latter part of the 1930s, it was decided that a review of the social insurance was necessary, and in 1938 a new parliamentary Inquiry (*Socialvårdskommittén*) was appointed by the Social democratic and Agrarian Party government. It presented its proposal for a universal health insurance in 1944.

#### 1944: a second attempt to introduce a universal health insurance

The Inquiry highlighted financial barriers for the population to access necessary medical care. The Inquiry mentioned that the rural population often refrained from seeking hospital care due to high travel costs, and although district doctors serving the rural population were relatively cheap, it was still economically burdensome for the less wealthy. The Inquiry stated that “From the public’s point of view, such measures [new solutions to ensure access] are necessary to protect people’s health and ability to work as far as possible” (44, p 115). Making it an insurance only for wage workers (as in some other countries) was regarded as an “unattractive” way of “making a distinction between different social groups” (44, p 134).

It was proposed that the insurance would cover “in principle all people residing in Sweden, from birth to death” (44, p 134). It consisted of two parts: (1) healthcare insurance and (2) sickness benefit insurance. Although it was not considered “an entirely satisfactory solution”, the main principle was that the insurance did not provide medical care but reimbursed peoples expenses for healthcare in case of illness (not preventive care, still leaving it to left it to the doctors to decide what illness is). For the individual this meant that he/she *had to pay the full cost at the point of care utilization*. The Inquiry noted that this meant that people sometimes could not (fully) use their insurance and waited too long to seek care, which worsened the possibility to treatment. Regarding hospital care, however, the practice was in many cases that the sickness funds paid the hospitals directly, thus increasing access for those with limited means.

The insurance was proposed to cover reimbursement for medical care (75% to prevent overuse), travel costs, reimbursement for certain medications, as well as in- and outpatient hospital care. The insurance was to be funded by fees (a flat rate) to a local public health insurance fund (in practice, the employer paid the fee and made a deduction from the salary) and by public funding.

#### 1944–1955: postponed reform due to disagreement and financial obstacles

The decade following the proposal in 1944 ended up in turmoil. The referral bodies were positive to the Inquiry’s proposal, but the then-Minister of Social Affairs, Social Democrat Gustav Möller instead presented a new compulsory health and medical insurance plan (inspired by the 1942 Beveridge Report) including a fully tax-funded healthcare system and a primarily tax-funded sickness benefit [43]. The idea was that healthcare should be a citizen right and not be dependent on previously paid fees, and contribute to income equalization [35]. Despite opposition from The Right, The Riksdag (Social Democrats, Communist Party, Agrarian Party and some members from the Liberal People’s Party) approved Möller’s proposal [45] with plans to implement it in 1950. However, an opposition was formed soon after the decision was made [35, 43]. The overheated economy, coupled with a shortage of doctors, nurses, and hospital beds, made it impossible to fund the reform through taxes, leading the Riksdag in 1948 to postpone the reform to 1951 [46]. In addition, other major reforms such as pensions and child allowance had recently been implemented (1946 and 1948, respectively) and both Social Democrats and the centre-right parties thought the reform would become too expensive [35]. In 1950, the Social Democratic prime minister declared that the reform was postponed until further notice [47]. When a new government was formed in late 1951, Möller was replaced by a new minister of Social Affairs, Social Democrat Gunnar Sträng, who appointed a new Inquiry (*Socialförsäkringsutredningen*) presenting a new proposal for a universal health insurance in 1952 [48].

During this decade, the Höjer Investigation was also presented, in which the controversial physician Axel Höjer suggested that in principle all healthcare should be free for the individual and that ambulatory care should be expanded and provided by public providers at primary care centres. The Höjer Investigation received a lot of criticism, in particular from the medical profession that found it to impinge on core interests of the medical profession (such as the fee-for-service principle), which made reforms impossible [31, 49]. Höjer’s ideas instead came to be implemented during the 1960s and 1970s.

#### 1955: the first universal health insurance comes into effect

The new bill in 1952 (which in large parts was similar to the proposal from 1944) suggested that practically all inhabitants would be included in the compulsory health insurance. From the age of 16, individuals would become members of a general sickness fund, before that they would be insured as children. Housewives would be insured independently rather than as family members. This was seen as an advantage in terms of equalizing different population categories [48, 50].

The new insurance proposal followed a similar structure to previous proposals, consisting of two parts: (1) healthcare insurance and (2) sickness benefit insurance. The Inquiry emphasized that all insured individuals would have *the same rights to the healthcare insurance benefits*. The insurance provided compensation for medical and hospital care as well as travel to and from doctor or hospital visits, up to a certain degree. However, it only compensated for 75% of the costs, with a maximum rate, thus embedding potential financial barriers for some individuals. A maximum fee was established for those less well-off living in rural areas with long travel distances [42]. Although it had been decided in 1946 that hospital care would be provided free of charge outside of the general insurance [45], hospital care was ultimately included because the costs would otherwise be too great for the state. The cost of the insurance would be shared by the insured (44%), employers (27%), and the state (29%). This would require tax increases, according to the proposal [50]. The fee was to be paid in connection with tax collection, deducted from the salaries of employed individuals [42]. By this reform, *a model with state-subsidized healthcare for all* was in place, thus introducing a system of universal health coverage.

### The era of emphasis on equity and public provision (1955–1989)

After the enactment of the universal health insurance, a consolidation phase began, and there was a period of cumulative public takeover of previously privately delivered services. The more pronounced emphasis on equity and equal access was articulated in the Seven Crowns Reform in 1970 (reducing financial barriers) and in the new healthcare legislation coming into force in 1983 (establishing a horizontal equity principle and counteracting personal barriers to access). During this time, Sweden had Social Democratic governments between 1932 and 1976 and from 1982 to 1991. In particular during the third quarter of the century (the golden age), Sweden accelerated its spending on health and on the training of doctors and the economy grew at express rate [49].

#### 1955–1969: responsibility is transferred to the county councils

The general trend during these years (which had already begun in the 1930s) encompassed two main aspects. The first was a gradual shift of responsibility for providing healthcare to the county councils (nowadays regions). After, for example, the assumption of responsibility for long-term care in the early 1950s, the taking over of district doctors in 1963 and mental care between 1963 and 1967, the county councils in practice had “total responsibility” for health services in their respective geographical areas, predominantly involving public provision. The second trend was an increasing transfer of responsibilities to the hospitals. During the 1950–1960 s, specialized central hospitals were introduced in all county councils, and smaller hospitals also became more specialized.

To counterbalance the hospital-centric approach, an Inquiry proposed in 1958 that the county councils assume responsibility for district doctors and strengthen ambulatory care, which was recommended in several government investigations spanning the 1940–1960s [51]. In 1969, the National Board of Health and Welfare introduced a program of principles for ambulatory care, giving rise to the concept of Primary Care Centres (*vårdcentral*), which subsequently developed into public primary care centers housing two or more physicians and other professionals. Essentially, the program from 1969 aligned with the suggestions put forth as early as 1948 by the Höjer Investigation [52], despite its contentious nature at the time [31, 49]. Large primary care centers with multi-disciplinary staff are still a distinctive feature of the Swedish healthcare system. Access to primary care doctors has, however, been a recurring problem, with long waiting times and a lack of continuity of care currently being the main issues. At the beginning of 2024, 88% of Swedes received a medical assessment (by a doctor or other licensed healthcare professional) within the three days specified in the national waiting time guarantee. In the region with the poorest access to primary care, the figure was 70% [53].

#### 1970: the seven-crowns reform

In 1967, an Inquiry (*Sjukförsäkringsutredningen*) concluded that the supply of hospital care could not match the increasing demand [54]. Similar to earlier reports (e.g [55]) an expansion of ambulatory care was argued for, both primary care and at hospitals. The report in 1967 served as a foundation for the government bill proposing to reform funding of outpatient care [56], but uncharacteristically, the bill was not based on a special inquiry and was not deliberated openly [57]. The bill put forward by a Social Democratic government suggested a simplified compensation system. Patients were to pay a fixed fee of 7 SEK (which led to the name ‘the seven-crowns reform’) for a doctor’s visit or 15 SEK for a home visit, which also included referrals for x-rays, laboratory tests etcetera. It was stated that one of the purposes of simplifying the compensation-system was to facilitate the expansion of publicly delivered healthcare. It was deemed essential for patients to know about the costs for their healthcare in advance. The proposal also aimed to promote economic equalization, as it was considered “important from a fairness perspective to equalize the difference in costs for different patients in outpatient care” and to provide greater insurance coverage for patients who require extensive and costly medical care [56, p. 34]. It has been described as an equality reform because it promoted economic equalization [58] and was part of a “policy thrust” by the Social Democrats, in which “equality policy” was a major component to maintain voters [57].

For patients, the reform brought about easier payment for open care visits and reduced costs for those who needed long-term and extensive care, thus *reducing financial barriers* and *unchosen unmet need*. However, it also brought a significant change for medical professionals. For instance, doctors were no longer allowed to have private practices at public hospitals. Most doctors became publicly employed, with regulated salaries and working hours [57]. This was part of the process of reinforcing the system of ambulatory healthcare provided by public providers, which was deemed an important political goal for the healthcare system [54]. The reform can be seen as a big step towards a Beveridge model of tax-funded healthcare with public providers, with increased political control over healthcare organization and funding to enhance equity and efficiency [58]. A regulation of physician incomes was symbolic for the Social Democrats to demonstrate their commitment to social equality [57].

#### 1983: the modern healthcare legislation and the first overall goal for healthcare

In 1979, an Inquiry (*Hälso- och sjukvårdsutredningen*) appointed by a Social Democratic government presented a completely new legislation for the health and medical care services [32]. It was a framework law applying to both public and private providers. The previous law focused mainly on hospital care [32]. The new law came into force in 1983 (Hälso- och sjukvårdslag 1982:763) and, unlike the previous legislation, also included provisions for preventive care. The principle of *equal access to all health and medical care* was a basic issue in the law proposal [31].

The Inquiry stated that welfare politics aimed to create good living conditions and the best possible quality of life for people: health being one important aspect. However, the Inquiry noted the absence of an “overall codified healthcare policy goal in Sweden” [32, p. 299] and claimed that “it was time to codify society’s ambitions to eliminate economic, social, knowledge-related, and geographical obstacles for individuals to use the care organization” [32, p. 412]. In the government bill, the goal was formulated: “The goal of the health services/healthcare is good health and healthcare on equal terms for the entire population” [59, p. 3]. It was a clear statement of horizontal equity. It was specified that the meaning of “equal terms” was that *all should, in principle, have the same opportunities to gain access healthcare*. Their opportunity should not be affected by such conditions as nationality, sex, age, education, ability to pay, cultural differences, ability to take initiative, the type of disease or the durability of the disease. Only the *need* for healthcare should be decisive for the extent and nature of the interventions/efforts. It was pointed out that for example social and psychological factors could limit people’s propensity to seek care, from which followed “an obligation to society to eliminate e.g. financial, social and geographical obstacles for the individual to receive care” [32, p. 278].

Specifications about who should be prioritized were not included in the law until 1997, when it was added that “*the one with the greatest need of healthcare should be given priority*” (SFS 1997:142). The latter formulation adds a vertical equity principle to Swedish healthcare. The addition was a way to ensure that ethical principles for prioritization suggested by a parliamentary Inquiry (*Prioriteringsutredningen*) would be applied in practice [60], sparked by an increased pressure on efficient use of resources.

#### The era of choice and privatization (1989-)

The overall trend during this era, which largely correspond to the retrenchment phase/’silver age’ in welfare state development, has been to again open up for private providers in primary care as well as specialised care and to increase the patient’s possibilities to choose healthcare provider, which were limited during the 1970s and 1980s. In part, this has been a response to *organizational barriers to access*, such as long waiting-times. However, also other types of policy trends can be noted during this era, for example the current policy approach to provide health services closer to the patient, which in Sweden e.g. has been formulated in terms of making the primary care centres the hub of the healthcare system. During this era, Sweden has been governed alternately by Social Democrats and centre-right coalitions (the latter 1991–1994; 2006–2014; 2022-). Most of the pro-competition and pro-choice reforms have been driven by the centre-right parties, but overall, the same type of policy shift has occurred in other European health systems during the same period [61]. During this era, it has been noted that the Swedish healthcare model is becoming less distinct than it was, and increasingly similar to health insurance models [62]. This can be described as a revised model based on tax-funding but with choice and competition elements [63, 64].

#### 1989–2010: contracting out and increased patient choice

During the 1990s, the healthcare system in Sweden faced mounting pressure leading to demands for cost efficiency. Healthcare expenses had risen, and the country was also hit hard by the economic crisis in 1990–1994, when there was a centre-right government, leading to rationalization and structural change in the public sector [65]. Alongside the calls for improved system efficiency, there were complaints about the system’s ‘bureaucraticness’, leading to demands for greater patient empowerment. Consequently, a series of reforms aimed at introducing private providers (through outsourcing and any-willing-provider systems) and enhancing patient choice were implemented between 1989 and 2010. Some aspects of this development have been contentious, such as the decisions to allow private companies to run acute care hospitals and university hospitals [66] and the introduction of free establishment for private companies in primary care [67]. Attempts have been made to reverse some of these reforms [68], but they have been unsuccessful. There have also been unsuccessful efforts to prohibit profit-making in publicly funded healthcare [69]. An inquiry initiated by a Social Democratic and Green Party-government in 2015 suggested that a surplus generated by private companies from public reimbursements should, as a general rule, be reinvested into the business where they arose, for example a primary care centre, and that profits could not exceed 7% of working capital. The centre-right parties and the Sweden Democrats made clear that they would not pass a bill that contained profit limitations. One of the leading Moderate Party politicians argued that it would impact negatively on care quality as competition is an important driver [70].

#### 2010: choice of primary care provider

In 2010, a reform was implemented that brought about significant changes in the planning, provision, and funding of primary care [71]. This was the result of an Inquiry (*Utredningen om patientens rätt*) appointed by the centre-right government entering office in 2006, with the task to suggest how to improve the patient’s position and how to regulate choice of primary care provider [72]. According to new law provisions, county councils were required to offer patients the freedom to choose their primary care provider, whether public or private. Private providers were granted the right to establish themselves and receive public funding under the same conditions as public providers.

The reform had two main objectives: empowering patients and facilitating the establishment of private primary care practices with public reimbursement [71]. The reform was argued to enhance the efficiency of care, improve access, and raise the quality of services. The then-Minister of Health and Social Affairs, Christian Democrat Göran Hägglund argued that the reform would lead to improved care for the individual and was bout “influence, quality, diversity [of providers], accessibility” [73]. However, opponents of the reform argued that it would likely favor those who were well-off and better equipped to make choices, *potentially undermining equal access*. Concerns were raised that primary care might become driven by demand rather than medical need, potentially neglecting the needs of the most vulnerable individuals [67]. Despite the potentially great impact on distribution of health resources in primary care, implications for equity were not systematically addressed by the policy-makers before implementing the reform. For example, the reform was not problematized in relation to existing inequalities in health access or outcomes [74].

#### 2020: challenging private health insurance

Since the 2000s, the number of Swedes with private health insurance has significantly risen, now covering about 14% of the employed population aged 15–74 [75]. These insurances can be classified as supplementary voluntary health insurance [76]. The primary benefit is quick access to healthcare, and this type of insurance is thus partly a response to the long waiting times. These insurances, which can be seen as *a response to organizational barriers to access*, primarily cover elective specialist care, with maximum waiting times of seven working days for specialist consultations and 14–21 working days for operations. In contrast, the publicly funded healthcare system guarantees a maximum wait time of 90 days for specialist consultations, and an additional 90 days if deemed necessary for operations. However, in practice, these limits are currently only attained for approximately 60–70% of patients. In some regions, however, the figure was below 50% at the beginning of 2024 [53].

The funding for private health insurance comes from insured individuals’ premiums (often a fringe benefit from the employer [77]), and they cover healthcare provided by contracted private providers. However, these providers can also have contracts with the public healthcare system/the regions, to offer care and treatment funded publicly by taxes (with the public maximum waiting-time limits). This arrangement has sparked a contentious debate about whether insurance patients receive priority over publicly funded patients [78, 79] *potentially violating the principle of “healthcare on equal terms”* as laid down in the Health and Medical Services Act (1982:763, updated 2017:30). Insurance companies argue that this is not the case and that private health insurance relieves the burden on the tax-funded healthcare system [80].

Consequently, differing viewpoints exist, which led the Social Democratic government to appoint a special investigator in 2020 to examine the impact of private health insurance on publicly funded healthcare and propose how to safe-guard equal healthcare [81]. It was first proposed that in order to prevent that private health insurance negatively affect ethical principles such as the needs principle, private providers contracted by a region to provide publicly funded healthcare *would not be allowed* to provide the same services or treatments to privately funded patients [82, p. 7–8]. The second proposal had another formulation [83]; when regions sign contracts with private healthcare providers who also have insurance companies as clients, it must be stated in the agreement *how it is ensured* that the private healthcare provider’s duties towards insurance patients do not have a negative impact on the care provided on behalf of the region. This was however downvoted in the Parliament in January 2023, which since September 2022 has a majority led by the Moderate Party-leader, Prime minister Ulf Kristersson. This means that there is no regulation of the impact of private health insurance on equal access to care, and currently a public system offering universal health coverage, and in parallel, a privately funded system that duplicates some services but provides quicker access.

## Discussion

As we have observed through the examination of macro-level policy developments over the past 100 years, Swedish healthcare has consistently evolved in terms of how the ideas of universal health coverage and equal access have been formulated. Despite being often regarded as a fixed model, Swedish healthcare has undergone successive transformation, with the period between 1970 and 1990 being particularly significant in shaping how the Swedish healthcare model is perceived. This model includes tax-funding, low out-of-pocket payments, the overarching goal of equal access for all, and predominantly public provision of services. The same phenomenon has been noted elsewhere: that the account of the Swedish welfare state and Swedish politics reflect “at best, times past when Sweden, according to some observers, stood out as an economic, political, and social success story in any international comparison [84]”. It is worth noting, however, that there was no universal health insurance until 1955, and the availability of own cash assets to cover direct payment at the point of care utilization was necessary up to a reform in the 1970s. This was the case even though the economic growth was unprecedented and a comprehensive welfare state was built [62, 63, 85]. Until 1983, Swedish healthcare did not have an overall codified policy goal and the pursuit of equal healthcare was thus more ideological.

In summary, during the first era, reforms aimed to establish a basic safety net to ensure that no individual would be deprived of medical treatment due to scarce resources. Following the implementation of the universal health insurance in 1955, efforts to equalize conditions for access peaked with the Seven Crowns Reform in 1970 and with the enactment of the new Health and Medical Services Act in 1983, which established equal access for all as the primary policy goal alongside the pursuit of good health for everyone. Subsequently, the pendulum has swung back, allowing for increased choice of provider, greater involvement of private providers, and privately funded solutions to enhance efficiency and access. Today, Sweden still rates high on affordability and equity in international comparisons of health system characteristics: however, not as good as ten years ago [22]. In 2022, Sweden spent a comparable percentage of GDP on health (10.7%) as countries such as Belgium, the Netherlands, Portugal, and Spain, but less than Austria, Canada, France, Germany, Japan, New Zealand, Switzerland, and the United Kingdom [86]. In the Commonwealth Fund’s comparison of performance of health care systems of eleven high-income countries, Sweden ranked just below the 10 country-average on health care system performance compared to spending. Overall, Sweden ranked seventh: sixth in terms of access and equity, and fifth in terms of healthcare outcomes [34].

In the following sections, the general observations from the analysis of the three eras are discussed, drawing upon two egalitarian goals in the context of health: (1) equality of access, and (2) equal treatment for equal need [22].

### Equality of access

Universal health coverage in the WHO: s meaning can be achieved in many ways. In Sweden, it is clear that the preferred solution up until recently was to create a uniform system based on social equality. In the proposals from 1919 to 1944 it was established that it was not a preferred route to make the universal health insurance only a wage worker insurance because it made an unwanted distinction between social groups, and initial exemptions of high-income groups and groups with a better health insurance from their employer had been withdrawn in the version of the universal health insurance that entered into force in 1955. In the 1955 insurance, house wives were also included in their own right, not as family members, with the aim of increasing social equality.

Throughout the one hundred years studied here, *financial barriers* (affordability) were continuously discussed and combatted until the 1970s. In the years before 1955, it was repeatedly argued that those with a financially weak position had problems paying for necessary medical care, i.e. there was a substantial *unchosen unmet need* [24]. Salaried workers were of particular concern being dependent on good enough health to be able to work to make ends meet. People living in rural parts of Sweden were also considered particularly exposed because of long (and costly) travels. However, even after 1955, there were still financial barriers. This originates from the construction of the insurance that did not provide care but reimbursed the cost for medical care afterwards. Until 1970, when the Seven Crowns Reform took place, people had to pay the full cost for medical care as well as travel expenses at the time of care utilization (some exceptions for less well-off living in rural areas). Furthermore, prices were not regulated and hard to foresee, which made some abstain from seeking necessary care, which worsened their opportunities to get well from a treatment. This changed with the reform in 1970 when it was decided that patients were to pay a flat-rate fee of 7 SEK for a doctor’s visit. It made a particular difference for those that needed extensive and expensive medical care, i.e. the most ill. This was described as an attempt at equalizing opportunities to seek care by equalizing costs. However, the same cost affects those with different financial positions differently [22] and some may still have experienced financial hardship seeking care, for example being unable to take time away from work and risk losing the salary. Yet, the Seven Crowns Reform is an important reason for Sweden ranking high on affordability in international comparisons. No one has to end up in debt in case of unexpected illness, which is a main driver for the WHOs work to support universal health coverage. Nevertheless, the discussion about financial barriers is prominent once again due to the rapid expansion of number of Swedes with a private health insurance, which not everyone can afford, in particular insurance bought individually and not co-funded by employers or unions.

During the first 50–60 years covered here, the discussion was largely about people ‘having access’, i.e. to create opportunities for people to use healthcare services, by reducing geographical variation and geographical barriers to access (availability and accommodation). After that there was more discussion on ‘getting access’, i.e. how individuals seek and utilize care (approachability and acceptability). *Personal barriers* to seeking healthcare were not treated in a structural manner before the Inquiry that formed the basis for the Health and Medical Services Act that came into force in 1983 [32]. This was also the first time Swedish healthcare got an overall codified healthcare policy goal. The Inquiry discussed what equal terms meant, and it was specified that the opportunity to gain access to healthcare should not be affected by such conditions as nationality, sex, age, education, ability to pay, cultural differences, ability to take initiative, the type of disease or the durability of the disease. Although it did not result in any specific law formulations (apart from emphasising that the individual should receive adequate and customized information), the Inquiry drew eyes to the fact that social, cultural and psychological factors could limit people’s propensity of seeking care (acceptability). The Inquiry made a rather far-reaching suggestion that society should remove social, financial and geographical obstacles for individuals to receive care through collaboration between healthcare services and other societal actors. In 2020, this is discussed again since not everyone can buy a private health insurance, e.g. because of age or previous illness, which constitutes a personal barrier.

*Organizational barriers* to access have been a recurring discussion over the past 100 years. In the early days it was primarily a matter of lack of health professionals and hospital beds and access barriers for the rural population (availability and accommodation). During many years, the preferred policy direction was to expand ambulatory care because the hospital sector was too costly. An attempt was made in 1948, then during the 1960–1970s, and currently through an extensive policy drive to bring care closer to the patient, with primary health care as the hub of the health system. A reorientation of health systems to primary health care is also recommended by the WHO to achieve universal health coverage, but Sweden has some way to go as a relatively small portion of healthcare expenditures are for primary care (∼ 18% of the regions’ costs in 2022). However, organizational barriers in the form of long waiting times to both primary care and specialist care is one of the main driving forces behind the development with an increasing share of Swedes with a private health insurance, besides ideological and economic positions. Timeliness of care has been described as the Achilles heel of health services in Sweden [87] and this has spurred privately funded solutions to quicker access. As in many other countries in Europe, they are still relatively small in terms of total of expenditure, well under 5% [76]. The employers and unions are a key player in this development and their rationale for providing and subsidizing a private health insurance for their employees is that they come back to work quicker if being ill or injured. To what extent private insurance correspond to unchosen unmet need in the population or subjective unmet expectations needs to be further investigated, but it seems as if those with a private health insurance are generally rather healthy [88]. However, due to the problem with long wait-times it could be argued that universal health coverage is not fully provided, as people do not have access to services “when they need them” [3].

### Equal treatment for equal need

In the literature, it is commonly described that health (and thereby healthcare) is needed for a person to flourish, to be able to realize his or her life plans. However, in 1919, when the first attempt to lay out a universal health insurance was made, this was not the reason why increased coverage was proclaimed: instead health was described as important to be able to work and thus be able to earn an income to make ends meet. This continued to be a rationale in 1944: “a necessity to protect people’s health and ability to work, as far as possible”. It was not only until the preparatory work for the new law in 1983 that it was mentioned that social policy should help create “best possible quality of life for people”. This shows a shift in the view of the human being and the relationship between the individual and the society.

In the early versions of the universal health insurance, need was spoken about in terms of necessity. It was established that medical treatment (not preventive care) had to be *necessary* to be covered by the insurance. How to define what was necessary, was however left to the doctors. Evidently there were concerns that not all medical care demanded would be sufficiently necessary and the decision to reimburse only 75% of the costs for healthcare visits was to hinder overuse of healthcare services, i.e. to hinder demand-driven use of services. However, it likely created unmet need due to financial barriers as well. The principle of equal treatment for equal need was actually not laid down in law until 1983, and not until 1997, it was established that “the one with the greatest need of healthcare should be given priority” (1997:142). Before that it was established that the goal was care on equal terms for the entire population (1982:763), which could be interpreted as the principle of horizontal equity: i.e. that “like needs should receive like attention and resources”. Thus, the principle of vertical equity, i.e. “that greater needs should receive greater attention and resources”, was thus not added until 1997 after a parliamentary Inquiry suggested the implementation of ethical principles for prioritization [60]. In tax funded health systems, these two principles together make up a redistribution of resources and health. The policy development during the third era (choice and privatization) has the potential of interfering with the principle of healthcare on equal terms (but according to need) because it is more demand-driven, although some other policy goals, such as increased access, may be to the benefit of the population. In fact, access to primary care has increased after the choice reform in 2010 [89], with nearly 300 new private primary care centres. More research is however needed to understand how increased access and utilization affect equity [90].

## Conclusion

The century-long perspective on macro-level policy with significance for universal health coverage and equal access in Sweden shows that different dimensions of access have been the focus of policy discussion, as well as how access should be made equal. The discussion of access barriers has moved from financial to personal and organizational ones during the previous century. Improving financial protection was undertaken step-wise, with the implementation of universal health insurance in 1955 and the introduction of a maximum direct payment at the point of care utilization in 1970. Explicit equity goals did not enter into legislation until 1983. Although few today abstain from seeking healthcare due to costs, long wait-times—organizational barriers—pose a challenge to achieving the conditions for universal health coverage, illustrating shifting challenges over time. This indicates that not only must the population be able to afford healthcare, the system must also deliver services on time in an equal manner to provide universal health coverage and equal access. In the future, we will be able to assess whether Sweden’s somewhat declining healthcare system outcomes mark the beginning of a new trend intertwined with a decline in Sweden’s welfare ‘exceptionalism’ [84], or if it is temporary.

## Data Availability

No datasets were generated or analysed during the current study.
